# The efficacy of deep learning models in the diagnosis of endometrial cancer using MRI: a comparison with radiologists

**DOI:** 10.1186/s12880-022-00808-3

**Published:** 2022-04-30

**Authors:** Aiko Urushibara, Tsukasa Saida, Kensaku Mori, Toshitaka Ishiguro, Kei Inoue, Tomohiko Masumoto, Toyomi Satoh, Takahito Nakajima

**Affiliations:** 1 Department of Radiology, Faculty of Medicine, University of Tsukuba, 1-1-1 Tennodai, Tsukuba, Ibaraki 305-8575 Japan; 2 Department of Diagnostic Radiology, Toranomon Hospital, 2-2-2 Toranomon, Minato-ku, Tokyo, 105-8470 Japan; 3 Department of Obstetrics and Gynecology, Faculty of Medicine, University of Tsukuba, 1-1-1 Tennodai, Tsukuba, Ibaraki 305-8575 Japan

**Keywords:** Endometrial carcinoma, Artificial intelligence, Convolutional neural network, CNN, Magnetic resonance imaging

## Abstract

**Purpose:**

To compare the diagnostic performance of deep learning models using convolutional neural networks (CNN) with that of radiologists in diagnosing endometrial cancer and to verify suitable imaging conditions.

**Methods:**

This retrospective study included patients with endometrial cancer or non-cancerous lesions who underwent MRI between 2015 and 2020. In Experiment 1, single and combined image sets of several sequences from 204 patients with cancer and 184 patients with non-cancerous lesions were used to train CNNs. Subsequently, testing was performed using 97 images from 51 patients with cancer and 46 patients with non-cancerous lesions. The test image sets were independently interpreted by three blinded radiologists. Experiment 2 investigated whether the addition of different types of images for training using the single image sets improved the diagnostic performance of CNNs.

**Results:**

The AUC of the CNNs pertaining to the single and combined image sets were 0.88–0.95 and 0.87–0.93, respectively, indicating non-inferior diagnostic performance than the radiologists. The AUC of the CNNs trained with the addition of other types of single images to the single image sets was 0.88–0.95.

**Conclusion:**

CNNs demonstrated high diagnostic performance for the diagnosis of endometrial cancer using MRI. Although there were no significant differences, adding other types of images improved the diagnostic performance for some single image sets.

## Background

Endometrial cancer is the sixth most common malignant disorder in women worldwide [1]. About 417,000 new cases of endometrial cancer were diagnosed worldwide in 2020, and about 97,000 people died from this disease [1]. The incidence of endometrial cancer is on the rise [2]. Surgery and biopsy are the standards for staging endometrial cancer, and MRI can assist in preoperative evaluation and surgical planning by accurately predicting the depth of invasion into the myometrium, invasion of the cervical stroma and surrounding organs, and the presence of lymph node metastases [3, 4]. Recently, multi-parametric MRI has been introduced to improve diagnosis [5]. In case the biopsy is not possible due to closure of the internal uterine ostium or no experience of sexual intercourse, MRI is also used to diagnose the presence of endometrial cancer [3]. Although MRI has not been formally incorporated into the FIGO staging system, it is already widely accepted as the most reliable imaging technique for diagnosing, staging, treatment planning, and follow-up of endometrial cancer. Moreover, MRI is said to minimize costs by eliminating the need for expensive diagnostic and surgical procedures [3].

In recent years, deep learning methods based on convolutional neural networks (CNN) have achieved remarkable performance in image pattern recognition [6, 7]. Moreover, a wide variety of computer vision tasks have been reported in the literature including deep learning-based segmentation [8–10], lesion detection [11, 12], and classification [13, 14]. The diagnostic modalities that were investigated include ultrasound, radiograph, CT, and MRI. The application of CNN to tumor images has the potential to be applied not only to image interpretation assistance but also to screening, prognosis estimation, and selection of optimal treatment methods, and we believe that tumor detection is the first step. However, to the best of our knowledge, no previous study has developed a CNN for diagnosing the presence of endometrial cancer. In addition, few studies have investigated optimal image conditions for MRI with multiple sequences and cross-sections in image classification using deep learning.

The present study constructed CNNs for diagnosing endometrial cancer using several sequences and cross-sections and its combination to validate for optimal CNN imaging conditions, and compared their diagnostic performance with that of experienced radiologists. Furthermore, we verified whether the diagnostic performance could be improved by the addition of sequences and cross-sections, other than the same type as the test image set, to the training data.

## Materials and methods

This retrospective study was approved by the Ethics Committee of University of Tsukuba Hospital (approval number: R02-054) and the requirement for written informed consent was waived. All methods were carried out in accordance with relevant guidelines and regulations.

### Study design

**Fig. 1:**
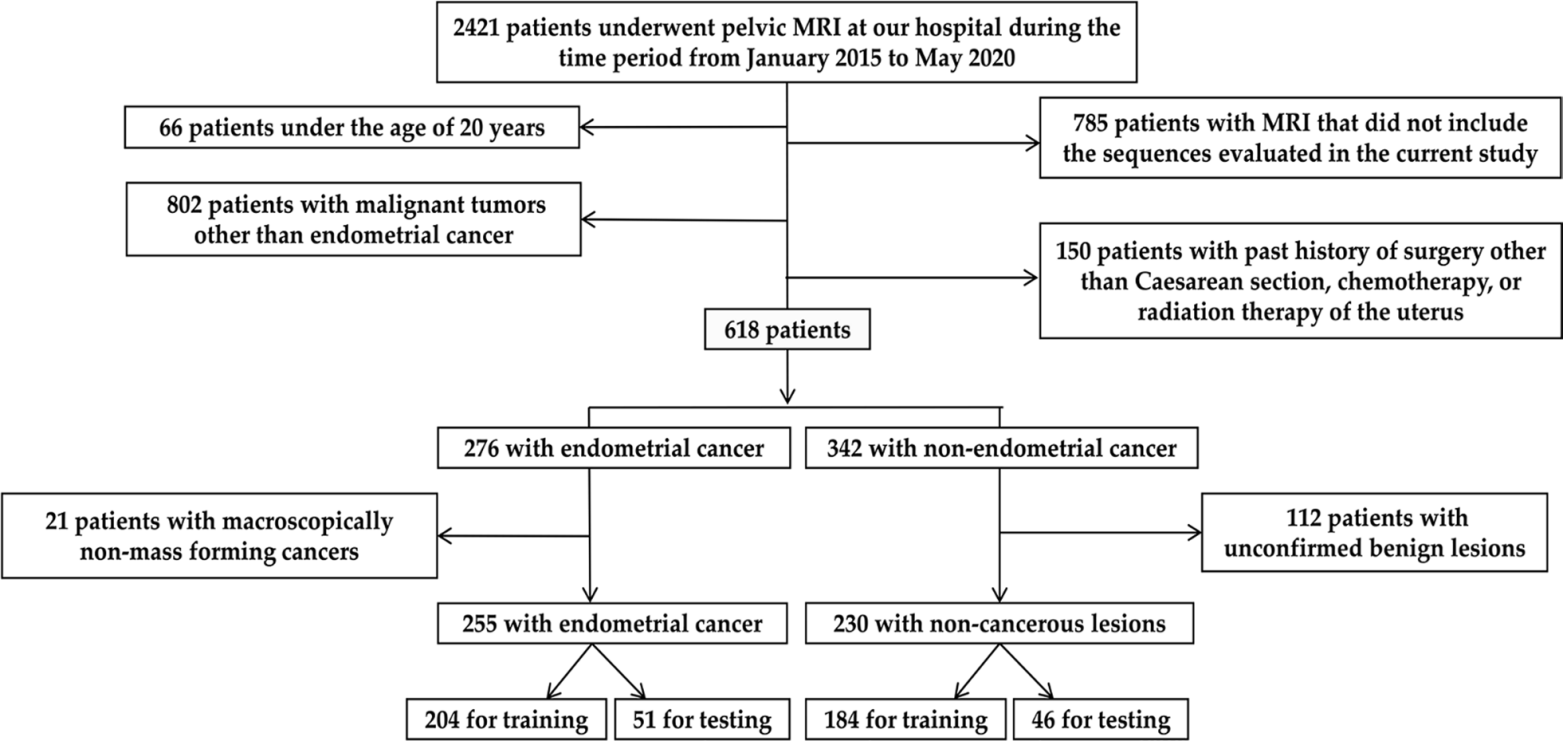
Flowchart of the patient selection process

The inclusion criteria are stated as follows: (A) woman above 20 years of age, (B) pelvic MRI scan obtained as per the protocol followed at our hospital during the time period from January 2015 to May 2020, (C) hysterectomized and pathologically confirmed as endometrial cancer (cancer group), and (D) pathologically or clinically benign lesions (non-cancer group). The exclusion criteria are stated as follows: (A) history of treatment for uterine diseases and (B) macroscopically non-mass-forming cancers according to pathological reports. A flowchart for the patient selection process is presented in Fig. 1.

**Fig. 2:**
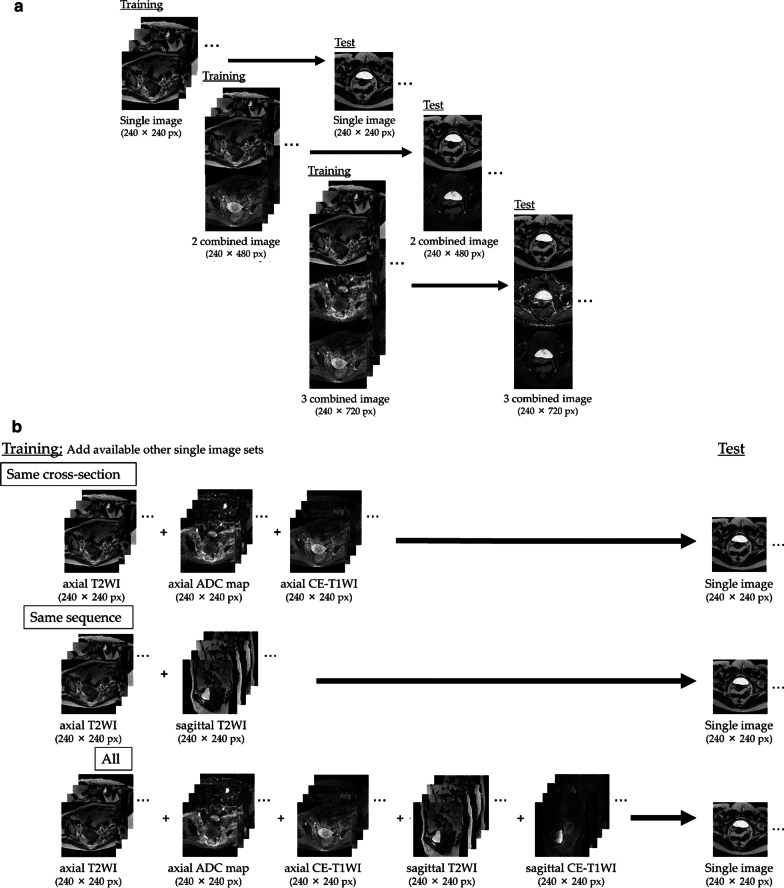
**a** Schematic diagrams of Experiment 1. **b** Schematic diagrams of Experiment 2. T2WI, T2 weighted image; ADC, Apparent Diffusion Coefficient; CE-T1WI, contrast-enhanced T1 weighted image

Figure 2 shows a flow diagram of the study design. As shown in Fig. 2a, Experiment 1 constructed CNNs for diagnosing the presence of endometrial cancer. Single and combined image sets of T2-weighted image (T2WI), apparent diffusion coefficient of water (ADC) map, and contrast-enhanced T1-weighted image (CE-T1WI) were used to validate optimal imaging conditions for CNN, and we compared their diagnostic performance with those of experienced radiologists. As shown in Fig. 2b, Experiment 2 verified whether the diagnostic performance could be improved by the addition of sequences and cross-sections, other than the same type as the test image set, to the training data.

### MRI acquisition

The MRI scan was performed using 3 T or 1.5 T equipment (Ingenia®, Achieva®; Philips Medical Systems, Netherlands) with a 32-channel phased-array body coil. The protocol employed to obtain the image of the entire uterus along the uterine axis included T2WIs, Diffusion-weighted images (DWIs) (b-value: 0, 1000), and CE-T1WIs of the equilibrium phase (Table 1). Gadopentetate dimeglumine 5 mmol (Magnevist® 0.5 mol/L or Gadovist® 1.0 mol/L; Bayer, Germany) was used for CE-T1WIs. The gadolinium dose varied according to the patient's weight, as recommended (0.2 ml/kg). Bolus intravenous contrast injection rate was 4 mL (2 mmol)/sec (in case of Gadovist, dilute with saline solution and inject at 4 ml/sec).

**Table 1:** MRI acquisition parameters

Scanner	Sequence	Cross-section	Type	TR/TE (ms)	FA (degree)	Slice/Gap (mm)	FOV (mm)	Matrix
Ingenia® 3.0 T	T2WI	Sg	2D-TSE	1400/110	90	3–5/0.3–0.5	280	640 × 640
	T2WI	Ax	2D-TSE	4955–5789/100–110	90	3–5/0.3–0.5	280	704 × 704
	DWI	Ax	EPI	6500–7500/77–79	90	3–5/0.3–0.5	280	224 × 224
	CE-T1WI	Sg	3D-GRE SPIR	4/2	10	3.3/1.6	280	576 × 576
	CE-T1WI	Ax	3D-GRE SPIR	4/2	10	3.3/1.6	280	576 × 576
Achiva® 1.5 T	T2WI	Sg	2D-TSE	1400/100–110	90	3–5/0.3–0.5	280	512 × 512–640 × 640
	T2WI	Ax	2D-TSE	1400–6013/100–110	90	3–5/0.3–0.5	280	512 × 512–704 × 704
	DWI	Ax	EPI	3963–7500/70–77	90	3–5/0.3–0.5	280	224 × 224–256 × 256
	CE-T1WI	Sg	3D-GRE SPIR	4–5/2	15	4.4/2.2	280	336 × 336–576 × 576
	CE-T1WI	Ax	3D-GRE SPIR	5/2	15	2/1	250–280	320 × 320–576 × 576

T2WI, T2 weighted image; DWI, Diffusion-weighted image; CE-T1WI, contrast-enhanced T1 weighted image; TR, repetition time; TE, echo time; FA, flip angle; FOV, field of view; Sg, sagittal; Ax, axial; TSE, turbo-spin echo;.EPI, echo planar imaging; GRE SPIR, gradient echo spectral pre-saturation with inversion recovery

### Data set

The image slices comprising the endometrium were extracted to create a dataset. In the cancer group, the sequences and pathological findings were considered and only the image slices depicting the tumor were visualized and extracted, as per the consensus of two radiologists (A.U., T.S.). The same cross-sectional images were extracted for all the sequences.

A total of 485 patients were randomly assigned to the training and testing groups. In the training phase, images obtained from 388 patients (204 and 184 patients in the cancer and non-cancer groups, respectively) were used; 2,905 axial images (1,471 and 1,434 images in the cancer and non-cancer groups, respectively) were used in each T2WI, ADC map, and CE-T1WI; 1,105 sagittal images (624 and 481 images in the cancer and non-cancer groups, respectively) were used in both T2WI and CE-T1WI. In the testing phase, only one central image of the stack was extracted, and 97 images (51 and 46 images from the cancer and non-cancer groups, respectively) were used in each sequence and cross-section.

The digital imaging and communications in medicine (DICOM) images were converted to joint photographic experts group (JPEG) images using the viewing software Centricity Universal Viewer (GE Healthcare, Chicago, Illinois, United States) because the graphical deep learning software we used could not handle the DICOM data itself. Subsequently, the JPEG images were resized to 240 × 240 pixels by trimming the margins using the XnConvert (Gougelet Pierre-Emmanuel in Reims, France), in order to perform the analysis. Along with the five single image sets, four combined image sets, including axial T2WI + ADC map, axial T2WI + CE-T1WI, sagittal T2WI + CE-T1WI, and axial T2WI + ADC map + CE-T1WI, were created for training and testing. The axial images were vertically combined (240 × 480 or 240 × 720 pixels) and the sagittal images were horizontally combined (480 × 240 pixels) using ImageMagick [15].

### Experiment 1: diagnostic performance for the single and combined image sets: CNN vs. radiologists

The current study compared the diagnostic performance of the CNNs and three board certificated radiologists with 27, 26, and 9 years of experience in pelvic MRI interpretation (T.M., K.M., and T.I.) using five single image sets and four combined image sets. The same types of single or combined image sets were used for training and testing. The radiologists were blinded to the clinical and pathological findings and independently reviewed the 97 randomly ordered test images in each image set, and reported the confidence levels in the presence of cancer using a 6-point scale (0, definitely absent; 0.2, probably absent; 0.4, possibly absent; 0.6, possibly present; 0.8, probably present;1.0, definitely present). The interpretation commenced with the single image sets (ADC map first), followed by the combined image sets. A time interval of one week was maintained between the sessions of interpretation.

### Experiment 2: CNN in testing the single image sets using different image sets for training

Experiment 2 investigated whether the addition of different types of image sets for training improved the diagnostic performance of CNNs. The CNN was trained using images of the same sequence regardless of the cross-sections, same cross-sectional images regardless of the sequences, and all images regardless of the sequences and cross-sections, in order to test five single image sets; only the single image sets were used for training and testing.

### Deep learning with convolutional neural networks

Deep learning was conducted on Deep Station Entry (UEI, Tokyo, Japan) with a GeForce RTX 2080Ti graphics processing unit (NVIDIA, Calif, USA), a Core i7-8700 central processing unit (Intel, Calif, USA), and the graphical deep learning software Deep Analyzer (GHELIA, Tokyo, Japan). The conditions optimized based on the ablation and comparative studies of the previous research were as follows: Xception [16], which is characterized as depth-wise separable convolutions that enable the use of model parameters more efficiently than the previous CNN architecture was used for deep learning, and ImageNet [17] which consists of natural images was used as pre-training. The parameters of optimization are stated as follows: optimizer algorithm = Adam (learning rate = 0.0001, β1 = 0.9, β2 = 0.999, eps = le-7, decay = 0, AMSGrad = false). The batch size was automatically selected. Horizontal flip, rotation (± 4.5°), shearing (0.05), and zooming (0.05) were automatically used as the data augmentation techniques. The CNNs were generated by setting the training/validation split ratio to 9:1, 8:2, or 7:3, and the epochs to 50, 100, 200, 500 or 1000 and the diagnostic results of each were validated. The training/validation split ratio and epochs were selected for each image set on the basis of the best performance among the CNNs with sensitivity and specificity above 0.75 (Table 2).

**Table 2:** The best settings for training/validation split ratio and epoch in Experiment 1 and 2

Test image set	Training image set	Training/validation split ratio	Epoch
**Experiment 1**			
Axial ADC map	Axial ADC map	9:1	100
Axial T2WI	Axial T2WI	9:1	50
Sagittal T2WI	Sagittal T2WI	8:2	50
Axial CE-T1WI	Axial CE-T1WI	8:2	200
Sagittal CE-T1WI	Sagittal CE-T1WI	8:2	100
Combined axial T2WI + ADC map	Combined axial T2WI + ADC map	9:1	100
Combined axial T2WI + CE-T1WI	Combined axial T2WI + CE-T1WI	9:1	100
Combined sagittal T2WI + CE-T1WI	Combined sagittal T2WI + CE-T1WI	9:1	50
Combined axial T2WI + ADC map + CE-T1WI	Combined axial T2WI + ADC map + CE-T1WI	9:1	200
**Experiment 2**			
Axial ADC map	All axial	8:2	50
Axial ADC map	All	9:1	50
Axial T2WI	All T2WI	8:2	100
Axial T2WI	All axial	9:1	50
Axial T2WI	All	9:1	50
Sagittal T2WI	All T2WI	8:2	200
Sagittal T2WI	All sagittal	8:2	200
Sagittal T2WI	All	8:2	100
Axial CE-T1WI	All CE-T1WI	8:2	50
Axial CE-T1WI	All axial	8:2	100
Axial CE-T1WI	All	8:2	100
Sagittal CE-T1WI	All CE-T1WI	9:1	100
Sagittal CE-T1WI	All sagittal	9:1	50
Sagittal CE-T1WI	All	9:1	100

ADC, Apparent diffusion coefficient; T2WI, T2 weighted image; CE-T1WI, contrast-enhanced T1 weighted image

### Statistical analysis

Statistical analyses were conducted using EZR (Saitama Medical Center, Jichi Medical University, Saitama, Japan), a graphical user interface for R (The R Foundation for Statistical Computing, Vienna, Austria), and SPSS software (SPSS Statistics 27.0; IBM, New York, NY, USA). The clinical values for each group were compared using the Mann–Whitney U test and the chi-square test. For the evaluation of the test data, in radiologists, 0.0–0.4 was treated as non-cancer and 0.6–1.0 was treated cancer. In CNN, the classification into cancer and non-cancer groups was output as a continuous number from 0 to 1, 0–0.49 was considered as non-cancer, and 0.50–1.0 was considered as cancer. The results were used to evaluate the sensitivity, specificity, and accuracy in cancer diagnosis. The receiver operating characteristic (ROC) analysis was performed to evaluate the diagnostic performance [18]. For statistics, 95% confidence intervals (CIs) and significant differences were estimated. Interobserver agreement was assessed with Kappa (κ) statistics [19]. *P* < 0.05 was considered to be significant.

## Results

### Patients and tumor characteristics from the training and test cohort

A total of 485 women (mean age, 52 years; age range, 21–91 years) were evaluated across the datasets. Table 3 shows the characteristics pertaining to the patients, the pathological types, and the number of each image. Although the patients in the cancer group were substantially older compared to the non-cancer group (*P* < 0.001), the present study did not observe any significant difference between the training and test data with respect to the age of the patients (*P* = 0.817). In the cancer group, 194 patients (train; 153, test; 41) were scanned with 3 T equipment, and 61 patients (train; 51, test; 10) were scanned with 1.5 T equipment. Also, in the non-cancer group, 166 patients (train; 131, test; 35) were scanned with 3 T equipment and 64 patients (train; 53, test; 11) were scanned with 1.5 T equipment. There was no significant difference in imaging equipment between the cancer group and the non-cancer group (train; *P* = 0.465, test; *P* = 0.789), and there was no significant difference in imaging equipment between training and testing (cancer; *P* = 0.533, non-cancer; *P* = 0.633). Of all, 55 patients in the non-cancer group (train; 47, test; 8) were clinically confirmed including imaging findings rather than pathological, and all others were pathologically confirmed.

**Table 3:** Characteristics of the patients and lesions

	Training data			Test data		
	Cancer	Non-cancer	All	Cancer	Non-cancer	All
**Patients (n)**	204	184	388	51	46	97
Age						
Mean (y ± SD)	58 ± 11.50	46 ± 12.00	52 ± 13.00	60 ± 13.74	44 ± 12.53	53 ± 15.44
Range (y)	28–83	22–81	22–83	30–91	21–71	21–91
**Pathological type (n)**						
***Benign (n)***						
Benign ovarian tumor		93			16	
Leiomyoma		71			24	
Endometrial hyperplasia		25			6	
Nabothian cyst		17			3	
Other		40			7	
**Malignant (n)**						
EC grade 1	118			30		
EC grade 2	51			11		
EC grade 3	20			9		
Other ECs	15			1		
Stage I	130			37		
Stage II	23			4		
Stage III	33			5		
Stage IV	18			5		
**Images (n)**						
Axial (T2WI, ADC map, CE-T1WI)	1,471	1,434	2,905	51	46	97
Sagittal (T2WI, CE-T1WI)	619	480	1,099	51	46	97

SD, standard deviation; EC, Endometrioid carcinoma; T2WI, T2 weighted image; ADC, Apparent Diffusion Coefficient; CE-T1WI, contrast-enhanced T1 weighted image

### Experiment 1

**Fig. 3:**
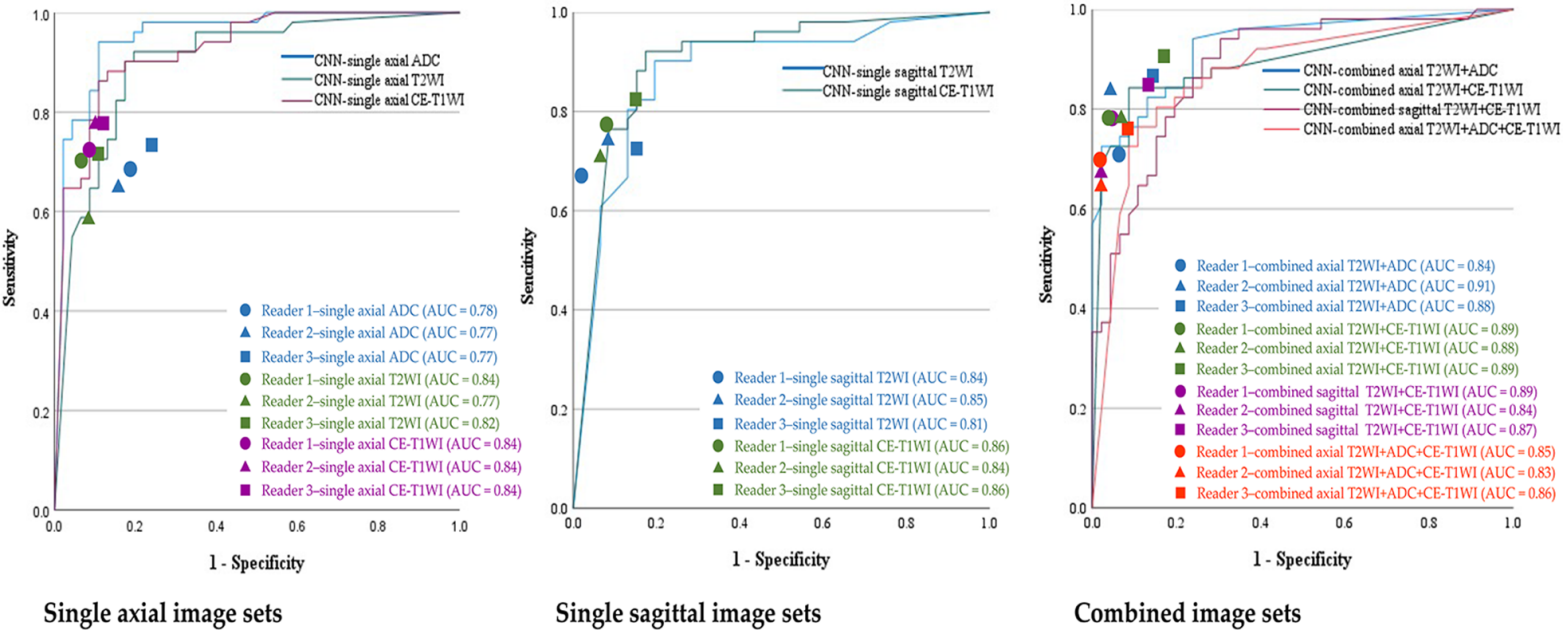
Experiment 1-The ROC curves for the CNNs. The ROC curves for the CNNs pertaining to the testing of the single and combined image sets with the AUC plots for the radiologists. T2WI, T2 weighted image; ADC, Apparent Diffusion Coefficient; CE-T1WI, contrast-enhanced T1 weighted image

**Fig. 4:**
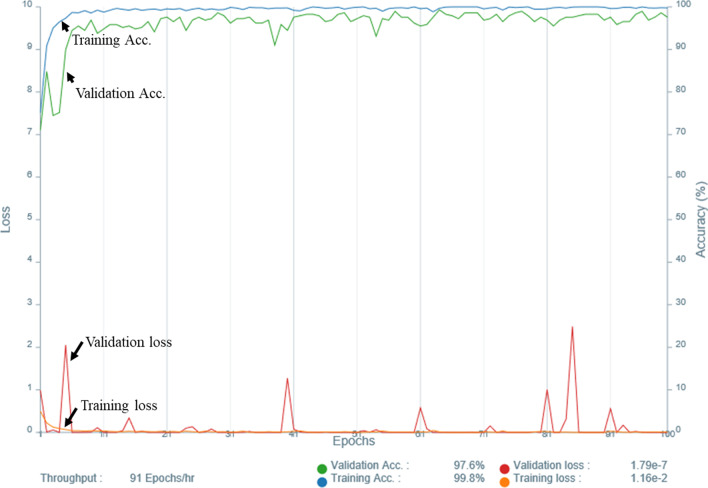
Accuracy and loss of the training data of the single image set of axial ADC map. Accuracy and loss of the training data of the single image set of axial ADC map with the training/validation split ratio 9:1 and epoch 100 in Experiment 1. Acc., accuracy

The results of Experiment 1 are presented in Table 4 and Fig. 3. Table 4 shows the diagnostic performance of the CNNs and radiologists for the single and combined image sets. Figure 3 shows the ROC curve comparing the performance of the CNNs for the single and combined image sets with the area under the receiver operating characteristic curve (AUC) pertaining to the radiologists. The sensitivity, specificity, accuracy, and AUC of the CNNs using the single and combined image sets were comparable to those displayed by the three radiologists. The AUC of the CNN was significantly higher for the single image sets of axial ADC map and axial CE-T1WI, compared to the three radiologists, and on the single image set of axial T2WI, compared to reader 2, and the combined image set of axial T2WI + ADC map, compared to reader 1. The present study did not observe any other significant difference between the CNNs and the three radiologists. The CNN showed the highest diagnostic performance with the single image set of axial ADC map with an AUC of 0.95. The graphs of accuracy and loss of the training data of the single image set of ADC map are shown in Fig. 4. The AUC of the CNNs for the combined axial T2WI + ADC map + CE-T1WI was 0.87, which was the lowest among the CNNs’ results for all the single and combined image sets.

**Table 4:** Experiment 1- Diagnostic performance of the CNNs and radiologists

Image set	Interpreter	Sensitivity	Specificity	Accuracy	AUC	*P*-value for AUC (vs. CNN)
Axial ADC map	CNN	0.94 (0.87–0.98)	0.87 (0.79–0.91)	0.91 (0.83–0.95)	0.95 (0.91–1.00)	–
	Reader1	0.71 (0.56–0.83)	0.85 (0.71–0.94)	0.77 (0.68–0.85)	0.78 (0.70–0.86)	< 0.001*
	Reader2	0.67 (0.52–0.79)	0.87 (0.74–0.95)	0.76 (0.67–0.84)	0.77 (0.69–0.85)	< 0.001*
	Reader3	0.77 (0.63–0.87)	0.78 (0.63–0.87)	0.77 (0.68–0.85)	0.77 (0.69–0.86)	< 0.001*
Axial T2WI	CNN	0.90 (0.83–0.95)	0.83 (0.74–0.88)	0.87 (0.79–0.92)	0.90 (0.84–0.96)	–
	Reader1	0.73 (0.58–0.84)	0.96 (0.85–1.00)	0.84 (0.75–0.90)	0.84 (0.77–0.91)	0.220
	Reader2	0.61 (0.46–0.74)	0.94 (0.82–0.99)	0.76 (0.67–0.84)	0.77 (0.70–0.85)	0.015*
	Reader3	0.73 (0.58–0.84)	0.91 (0.79–0.98)	0.81 (0.72–0.89)	0.82 (0.75–0.89)	0.100
Sagittal T2WI	CNN	0.90 (0.82–0.95)	0.80 (0.72–0.86)	0.86 (0.77–0.91)	0.88 (0.81–0.95)	–
	Reader1	0.69 (0.54–0.81)	1.00 (0.89–1.00)	0.84 (0.75–0.90)	0.84 (0.78–0.91)	0.457
	Reader2	0.77 (0.63–0.87)	0.94 (0.82–0.99)	0.85 (0.76–0.91)	0.85 (0.78–0.92)	0.574
	Reader3	0.75 (0.60–0.86)	0.87 (0.74–0.95)	0.80 (0.71–0.88)	0.81 (0.73–0.89)	0.167
Axial CE-T1WI	CNN	0.84 (0.71–0.93)	0.89 (0.76–0.96)	0.87 (0.78–0.93)	0.93 (0.87–0.98)	–
	Reader1	0.75 (0.60–0.86)	0.94 (0.82–0.99)	0.84 (0.75–0.90)	0.84 (0.77–0.91)	0.006*
	Reader2	0.77 (0.63–0.87)	0.91 (0.79–0.98)	0.84 (0.75–0.90)	0.84 (0.77–0.91)	0.002*
	Reader3	0.77 (0.63–0.87)	0.91 (0.79–0.98)	0.84 (0.75–0.90)	0.84 (0.77–0.91)	0.014*
Sagittal CE-T1WI	CNN	0.90 (0.83–0.95)	0.83 (0.74–0.88)	0.87 (0.79–0.92)	0.90 (0.84–0.97)	–
	Reader1	0.78 (0.65–0.89)	0.94 (0.82–0.99)	0.86 (0.77–0.92)	0.86 (0.79–0.93)	0.336
	Reader2	0.73 (0.58–0.84)	0.96 (0.85–1.00)	0.84 (0.75–0.90)	0.84 (0.77–0.91)	0.173
	Reader3	0.84 (0.71–0.93)	0.87 (0.74–0.95)	0.86 (0.77–0.92)	0.86 (0.79–0.93)	0.341
Combined axial T2WI + ADC map	CNN	0.82 (0.69–0.92)	0.87 (0.74–0.95)	0.85 (0.76–0.91)	0.93 (0.88–0.98)	–
	Reader1	0.73 (0.58–0.84)	0.96 (0.85–1.00)	0.84 (0.75–0.90)	0.58 (0.48–0.68)	< 0.001*
	Reader2	0.84 (0.71–0.93)	0.98 (0.89–1.00)	0.91 (0.83–0.96)	0.91 (0.86–0.97)	0.598
	Reader3	0.88 (0.76–0.96)	0.87 (0.74–0.95)	0.88 (0.79–0.93)	0.88 (0.81–0.94)	0.196
Combined axial T2WI + CE-T1WI	CNN	0.84 (0.71–0.93)	0.91 (0.79–0.98)	0.88 (0.79–0.93)	0.89 (0.83–0.96)	–
	Reader1	0.80 (0.67–0.90)	0.98 (0.89–1.00)	0.89 (0.81–0.94)	0.89 (0.83–0.95)	0.943
	Reader2	0.80 (0.67–0.90)	0.96 (0.85–1.00)	0.88 (0.79–0.93)	0.88 (0.82–0.94)	0.720
	Reader3	0.92 (0.81–0.98)	0.85 (0.71–0.94)	0.89 (0.81–0.94)	0.89 (0.82–0.95)	0.839
Combined sagittal T2WI + CE-T1WI	CNN	0.94 (0.84–0.99)	0.74 (0.59–0.86)	0.85 (0.76–0.91)	0.89 (0.82–0.95)	–
	Reader1	0.80 (0.67–0.90)	0.98 (0.89–1.00)	0.89 (0.81–0.94)	0.89 (0.83–0.95)	0.890
	Reader2	0.69 (0.54–0.81)	1.00 (0.89–1.00)	0.84 (0.75–0.90)	0.84 (0.78–0.91)	0.375
	Reader3	0.86 (0.74–0.94)	0.87 (0.74–0.95)	0.87 (0.78–0.93)	0.87 (0.80–0.94)	0.667
Combined axial T2WI + ADC map + CE-T1WI	CNN	0.80 (0.67–0.90)	0.80 (0.66–0.91)	0.80 (0.71–0.88)	0.87 (0.80–0.94)	–
	Reader1	0.71 (0.56–0.83)	1.00 (0.89–1.00)	0.85 (0.76–0.91)	0.85 (0.79–0.92)	0.675
	Reader2	0.67 (0.52–0.79)	1.00 (0.89–1.00)	0.83 (0.73–0.89)	0.83 (0.77–0.90)	0.406
	Reader3	0.78 (0.65–0.89)	0.94 (0.82–0.99)	0.86 (0.77–0.92)	0.86 (0.79–0.93)	0.813

Diagnostic performance of the CNNs and radiologists in the test using the single and combined image sets

T2WI, T2 weighted image; ADC, Apparent Diffusion Coefficient; CE-T1WI, contrast-enhanced T1 weighted image, AUC, area under the receiver operating characteristic curve; Data in parentheses are 95% confidence interval. *P < 0.05

**Fig. 5:**
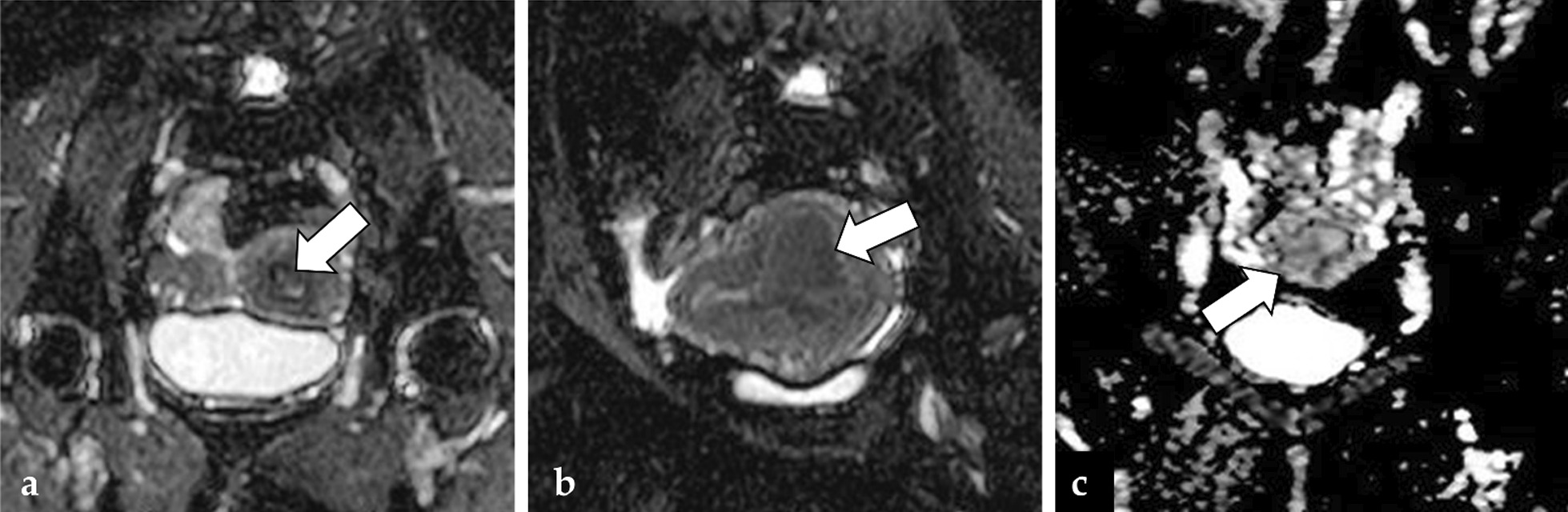
Three cases of false negatives were observed in the single image set of axial ADC: **a** A 55-year-old woman with grade 1 endometrioid carcinoma, in which the CNN was able to diagnose cancer, but the readers 1, 2, and 3 were not (the CNN confidence; cancer = 99.9%). The image shows a tiny tumor filling the uterine cavity (arrow); **b** A 34-year-old woman with grade 1 endometrioid carcinoma, in which all the three readers could diagnose cancer, but the CNN could not (the CNN confidence; cancer = 18.8%). The image shows a massive tumor protruding into the myometrium of the posterior wall of the uterus (arrow); **c** A 31-year-old woman with grade 2 endometrioid carcinoma, in which neither the CNN nor the three readers could diagnose the presence of cancer (the CNN confidence; cancer = 22.5%). The image shows the tumor filling the uterine cavity (arrow). A slight decrease in the single image of ADC map might have made the diagnosis of tumor difficult with a single image without considering the other images for radiologists

**Fig. 6:**
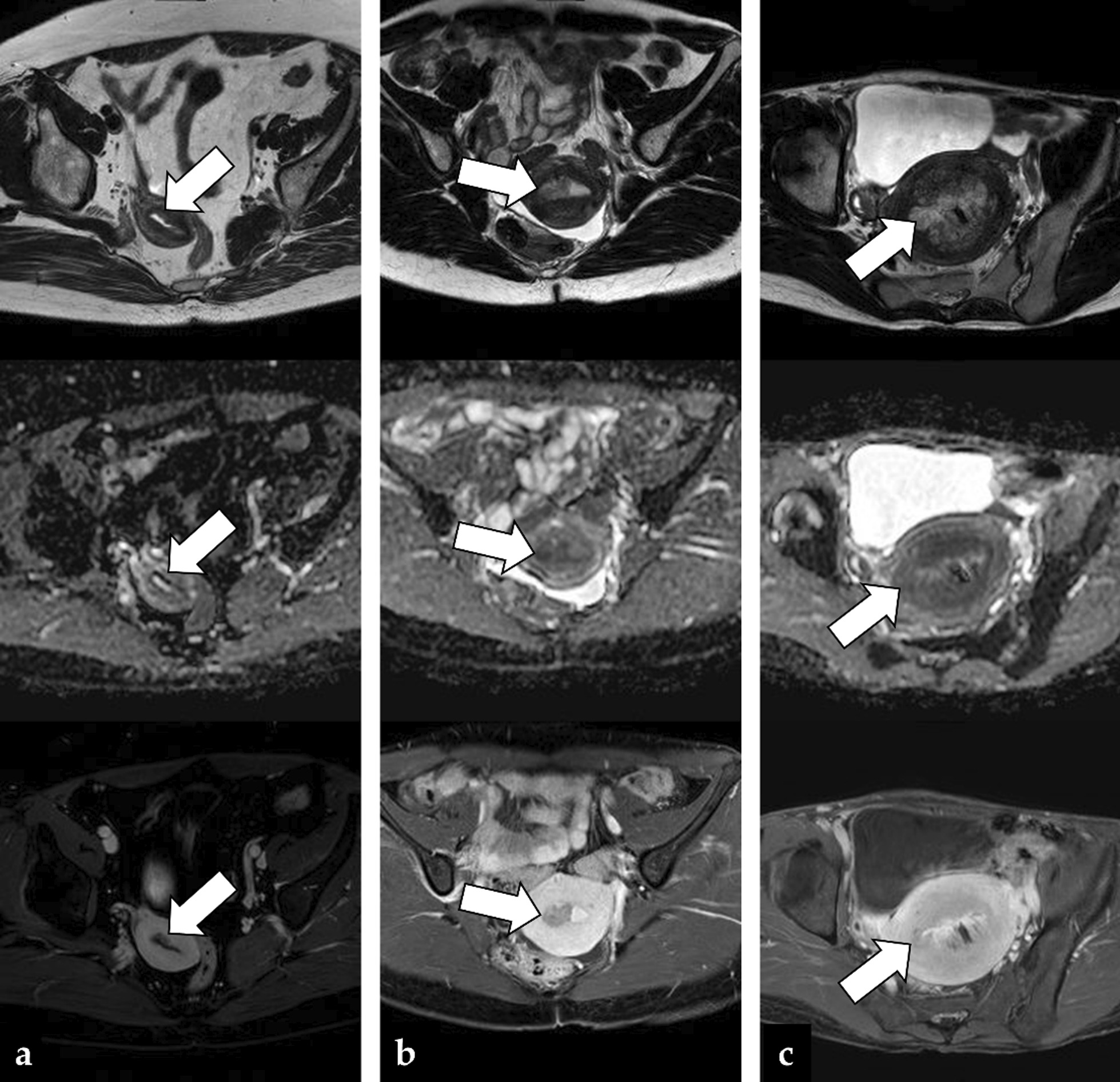
Three cases of false negatives were observed in the combined image set of axial T2WI + ADC + CE-T1WI: **a** A 56-year-old woman with grade 1 endometrioid carcinoma, in which the CNN was able to detect the cancer, but the three readers were not (the CNN confidence; cancer = 100%); **b** A 30-year-old woman with grade 1 endometrioid carcinoma, in which the three readers could diagnose the presence of cancer, but the CNN could not (the CNN confidence; cancer = 0.5%). The image shows a tumor displaying the typical appearance of endometrial cancer and filling the right side of the uterine cavity (arrow); **c** A 45-year-old woman with grade 1 endometrioid carcinoma, in which neither the CNN nor the three readers could diagnose the presence of cancer (the CNN confidence; cancer = 0.5%). The image shows a massive tumor filling the uterine cavity (arrow) and a hemorrhage at the center of the lesion. Non-uniform signal intensities of the tumor mass may have made the diagnosis difficult for radiologists

Figure 5 shows three false-negative cases reported by the radiologists (Fig. 5a), the CNN (Fig. 5b), and both the radiologists and the CNN (Fig. 5c) in the interpretation of the single iamge set of axial ADC map. Figure 6 shows three false-negative cases reported by the radiologists (Fig. 6a), the CNN (Fig. 6b), and both the radiologists and the CNN (Fig. 6c) in the interpretation of the combined image set of axial T2WI + ADC map + CE-T1WI. The confidence levels of the CNN in the diagnosis of endometrial cancer are shown in the figure legends for each case.

Table 5 shows the inter-observer agreement between the CNNs and the three radiologists. The ks between the CNN and radiologists ranged from 0.32–0.81, varying widely and were less consistent than those among the radiologists.

**Table 5:** Interobserver agreement between the CNN and the radiologists

		Reader 1	Reader 2	Reader 3
Axial ADC	CNN	0.410 (SD0.090)	0.515 (SD0.083)	0.443 (SD0.091)
	Reader2	0.558 (SD0.085)	–	–
	Reader3	0.629 (SD0.078)	0.609 (SD0.079)	–
Axial T2WI	CNN	0.496 (SD0.083)	0.322 (SD0.087)	0.493 (SD0.084)
	Reader2	0.584 (SD0.085)	–	–
	Reader3	0.702 (SD0.073)	0.546 (SD0.086)	–
Sagittal T2WI	CNN	0.483 (SD0.080)	0.453 (SD0.087)	0.450 (SD0.088)
	Reader2	0.722 (SD0.071)	–	–
	Reader3	0.598 (SD0.081)	0.624 (SD0.080)	–
Axial CE-T1WI	CNN	0.690 (SD0.073)	0.773 (SD0.064)	0.690 (SD0.073)
	Reader2	0.874 (SD0.050)	–	–
	Reader3	0.832 (SD0.057)	0.833 (SD0.057)	–
Sagittal CE-T1WI	CNN	0.654 (SD0.074)	0.576 (SD0.078)	0.567 (SD0.083)
	Reader2	0.831 (SD0.057)	–	–
	Reader3	0.794 (SD0.061)	0.712 (SD0.070)	–
Combined axial T2WI + ADC	CNN	0.335 (SD0.055)	0.412 (SD0.054)	0.339 (SD0.055)
	Reader2	0.727 (SD0.070)	–	–
	Reader3	0.714 (SD0.069)	0.774 (SD0.063)	–
Combined axial T2WI + CE-T1WI	CNN	0.814 (SD0.059)	0.752 (SD0.067)	0.651 (SD0.076)
	Reader2	0.853 (SD0.053)	–	–
	Reader3	0.756 (SD0.064)	0.695 (SD0.071)	–
Combined sagittal T2WI + CE-T1WI	CNN	0.604 (SD0.081)	0.453 (SD0.089)	0.609 (SD0.080)
	Reader2	0.807 (SD0.060)	–	–
	Reader3	0.836 (SD0.055)	0.693 (SD0.069)	–
Combined axial T2WI + ADC + CE-T1WI	CNN	0.550 (SD0.081)	0.469 (SD0.084)	0.486 (SD0.088)
	Reader2	0.777 (SD0.067)	–	–
	Reader3	0.724 (SD0.070)	0.765 (SD0.065)	–

ADC, Apparent Diffusion Coefficient; T2WI, T2 weighted image; CE-T1WI, contrast-enhanced T1 weighted image; SD, standard deviations

### Experiment 2

**Fig. 7:**
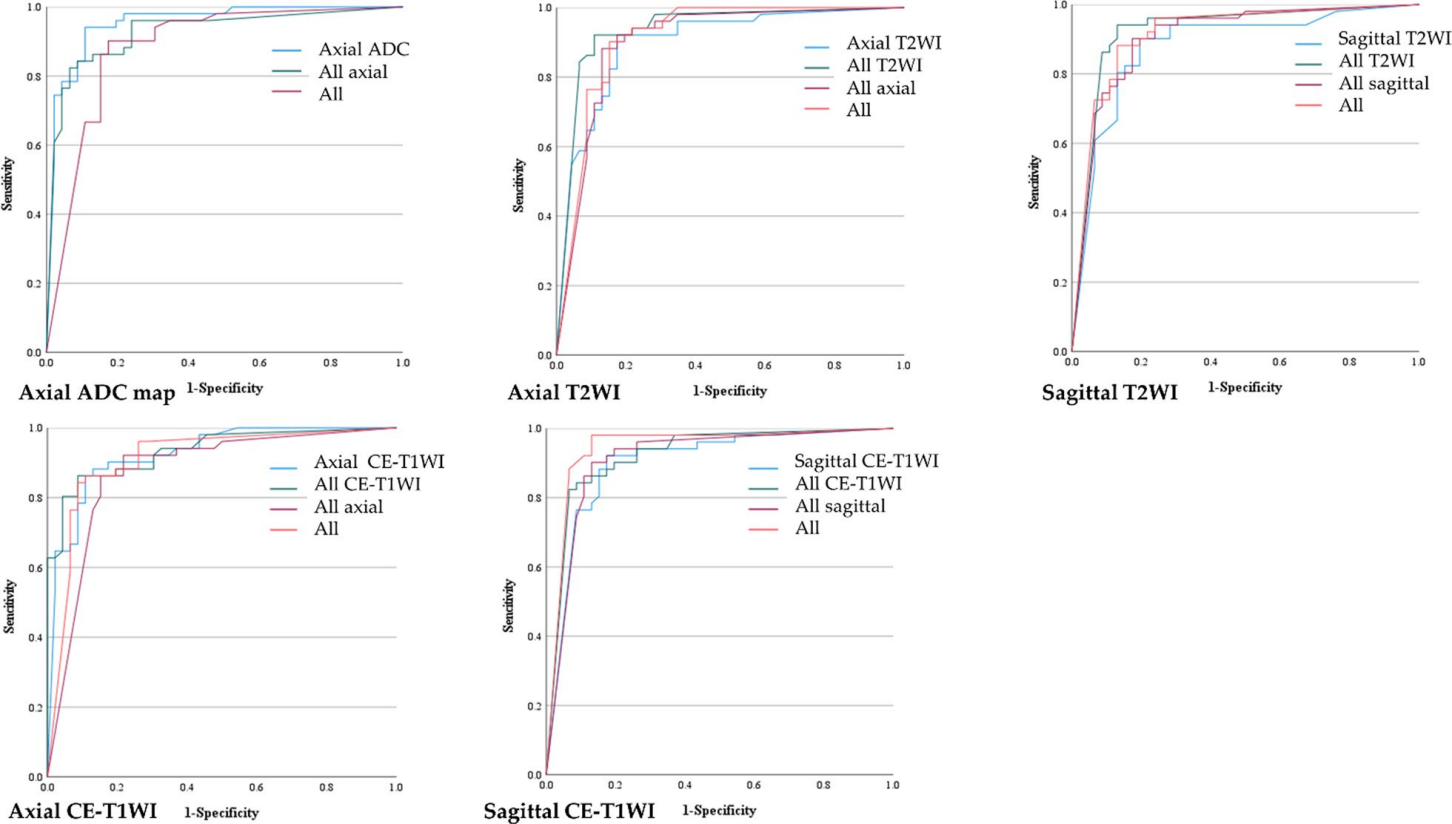
Experiment 2—The ROC curves for the CNNs. The ROC curves for the CNNs pertaining to testing the single image sets with various types of image sets for training. ADC, Apparent Diffusion Coefficient; T2WI, T2 weighted image; CE-T1WI, contrast-enhanced T1 weighted image

The results of Experiment 2 are presented in Table 6 and Fig. 7. Table 6 shows the diagnostic performance of the CNNs in testing using the single image sets and the addition of various types of image sets of different sequences and/or cross-sections to the training data. In this study, the AUC showed an increase when any types of image sets were added for training in the image set of sagittal T2WI and sagittal CE-T1WI, and all T2WI and all image sets were used for training in the image set of axial T2WI, although the difference was not significant. Conversely, for the image set of axial ADC map and axial CE-T1WI, the addition of any image set for training did not improve the AUC.

**Table 6:** Experiment 2-diagnostic performance of the CNNs

Test image set	Training image set	Sensitivity	Specificity	Accuracy	AUC	*P*-value for AUC^†^
Axial ADC map	Axial ADC map	0.94 (0.87–0.98)	0.87 (0.79–0.91)	0.91 (0.83–0.95)	0.95 (0.91–1.00)	–
	All axial	0.84 (0.76–0.90)	0.87 (0.78–0.93)	0.86 (0.77–0.91)	0.93 (0.87–0.98)	0.345
	All	0.90 (0.82–0.95)	0.80 (0.72–0.86)	0.86 (0.77–0.91)	0.89 (0.81–0.96)	0.069
Axial T2WI	Axial T2WI	0.90 (0.83–0.95)	0.83 (0.74–0.88)	0.87 (0.79–0.92)	0.90 (0.84–0.96)	–
	All T2WI	0.92 (0.85–0.96)	0.89 (0.81–0.94)	0.91 (0.83–0.95)	0.94^†^ (0.88–0.99)	0.218
	All axial	0.86 (0.79–0.91)	0.87 (0.78–0.93)	0.87 (0.78–0.92)	0.90 (0.84–0.97)	0.934
	All	0.90 (0.83–0.95)	0.85 (0.76–0.90)	0.88 (0.80–0.93)	0.91^†^ (0.85–0.98)	0.627
Sagittal T2WI	Sagittal T2WI	0.90 (0.82–0.95)	0.80 (0.72–0.86)	0.86 (0.77–0.91)	0.88 (0.81–0.95)	–
	All T2WI	0.94 (0.87–0.98)	0.80 (0.72–0.85)	0.88 (0.80–0.92)	0.92^†^ (0.86–0.98)	0.188
	All sagittal	0.90 (0.83–0.95)	0.83 (0.74–0.88)	0.87 (0.79–0.92)	0.91^†^ (0.84–0.97)	0.507
	All	0.86 (0.79–0.91)	0.87 (0.78–0.93)	0.87 (0.78–0.92)	0.92^†^ (0.85–0.98)	0.424
Axial CE-T1WI	Axial CE-T1WI	0.84 (0.71–0.93)	0.89 (0.76–0.96)	0.87 (0.78–0.93)	0.93 (0.87–0.98)	–
	All CE-T1WI	0.84 (0.77–0.89)	0.91 (0.83–0.96)	0.88 (0.80–0.92)	0.93 (0.89–0.98)	0.716
	All axial	0.92 (0.85–0.97)	0.78 (0.70–0.83)	0.86 (0.78–0.90)	0.88 (0.80–0.95)	0.086
	All	0.86 (0.78–0.92)	0.84 (0.76–0.91)	0.86 (0.77–0.91)	0.91 (0.85–0.97)	0.589
Sagittal CE-T1WI	Sagittal CE-T1WI	0.90 (0.83–0.95)	0.83 (0.74–0.88)	0.87 (0.79–0.92)	0.90 (0.84–0.97)	–
	All CE-T1WI	0.86 (0.79–0.91)	0.87 (0.78–0.93)	0.87 (0.78–0.92)	0.92^†^ (0.87–0.98)	0.524
	All sagittal	0.90 (0.83–0.95)	0.85 (0.76–0.90)	0.88 (0.80–0.93)	0.91^†^ (0.85–0.98)	0.696
	All	0.98 (0.92–1.00)	0.83 (0.76–0.84)	0.91 (0.84–0.92)	0.95^†^ (0.89–1.00)	0.156

Diagnostic performance of the CNNs in the testing using single image sets with the addition of other image sets for training

ADC, Apparent Diffusion Coefficient; T2WI, T2 weighted image; CE-T1WI, contrast-enhanced T1 weighted image

^†^ vs. the CNN trained with the single image set

## Discussion

Compared to the radiologists, the CNNs displayed non-inferior diagnostic performance in interpreting all five single image sets and significantly better results with the single image set of axial ADC map and axial CE-T1WI. Although there were no significant differences, the diagnostic performance improved by adding other types of image sets to the training data, except for the single image set of axial ADC map and axial CE-T1WI. The improvement in the interpretation of the combined image sets was not equivalent to that of the radiologists.

Several CNNs using MRI have been constructed to diagnose uterine tumors to date [20, 21]. Urushibara et al. recently developed a CNN that can differentiate between cervical cancer and non-cancerous lesions on T2WI [22]. Chen et al. and Dong et al. evaluated the myometrial infiltration of endometrial cancer using CNN and T2WI [23], and T2WI + CE-T1WI [24]. As far as we know, this is the first study to diagnose the presence of endometrial cancer and to assess the effects of adding other types of images to the training data and the conditions suitable for the application of deep learning in tumor classification. It is also noteworthy that the entire pelvic images were used, not just the cropped images of the uterus.

CE-T1WI and DWI are important sequences that allow the functional evaluation of endometrial cancer, and are clinically used as an adjunct to T2WI. The degree of tumor enhancement depends on the tumor vascularity; most endometrial cancers are hypovascular, while quite a few are isovascular or hypervascular, compared to the myometrium [25]. ADC values are inversely correlated to the tumor cellularity [26], and ADC values of endometrial cancer are significantly lower than endometrial polyps and normal endometrium [27, 28]. Hence, referencing CE-T1WI and ADC maps with T2WI improves cancer diagnosis. The present study observed that the CNNs displayed the best performance with the single image set of axial ADC map in Experiment 1, which is consistent with a previous study regarding the diagnosis of prostate cancer. The perception of anatomical structures using ADC maps alone is challenging for the radiologists. In contrast, ADC maps are considered to be suitable for cancer detection using CNN, and showing high diagnostic performance on ADC maps with low spatial resolution alone may be one of the CNN’s strengths. Conversely, it may be possible to improve the diagnostic performance of CNNs by increasing the number of training images even when using high-resolution images such as T2WI. Contrary to the current results, Aldoj et al. reported that the best diagnostic performance of the CNN was attained by combining ADC map + DWI + perfusion + T2WI [29]. This research differs from the present study in that a large number of (approximately 120,000) images were used for training. As the number of images to be combined increases, the variation in information also increases. Consequently, increasing the number of images used for training may be warranted.

The interobserver agreement between the CNNs and the radiologists tended to be lower than among the radiologists, and CNN may have used a different perspective than the radiologists and, therefore, may have made a completely different assessment. For the single image set of CE-T1WI or the combined image sets including CE-T1WI, interobserver agreement was high both between the radiologists and between the CNNs and the radiologists. The finding that the contrast effect of endometrial cancer on CE-T1WI is lower than the contrast effect of the myometrium is thought to make it easier for both radiologists and CNN to diagnose the presence of cancer.

Adding other types of image sets to the training data improved the diagnostic performance, except for the single image set of axial ADC map and axial CE-T1WI in Experiment 2. This result is similar to the recent report by Lee et al. that training with all available MRI sequences of the same cross-section improves the diagnostic performance of CNNs in distinguishing between pseudo and true tumor progression [30]. The present study observed that the addition of other cross-sections of the same sequence was especially beneficial. The amount of training data for the sagittal sections was smaller than the axial sections. Hence, the impact of the improvement may be greater. It is presumed that similar signal information is included in the same sequence even in different cross-sections, and similar morphological information is included in the same cross-section, even in different sequences. The potential for improved diagnostic performance by adding different sequences and cross-sections is an important result concerning the deep learning studies of tumor diagnosis, which involve difficulties in obtaining a large number of images. In order to establish the optimum image conditions in deep learning using MRI with various sequences and cross-sections, it is necessary to verify further using various combinations of various images in various regions.

The current study has several limitations. First, only one selected image was evaluated, which differs from the clinical practice of diagnosis using a series of images. It also differs from a clinical setting in that the JPEG images, which contain less information than DICOM images, were used. Second, the non-cancer group included lesions that were not pathologically confirmed. However, we considered it important to distinguish cancer from benign lesions that do not warrant treatment. Third, it is controversial whether atypical endometrial hyperplasia should be classified as benign because it is not cancerous or malignant because it is a precursor lesion. However, it would be unreasonable to exclude only atypical endometrial hyperplasia from this study. Therefore, in this study, we classified atypical endometrial hyperplasia as benign because the purpose was to detect endometrial cancer. Fourth, we have not examined dynamic studies to avoid study complexity. Although the dynamic study is useful to determine the degree of myometrial invasion, the contrast between the tumor and the myometrium is greatest during the equilibrium phase [3]. This study targeted the presence of cancer, so only the images of the equilibrium phase were used as contrast images. The following can be considered future improvements: the superiority of combined images may be demonstrated using more training data. The performance can be improved using three-dimensional images instead of two-dimensional images, as reported by Mehrtash et al., who used three-dimensional prostate images for convolutional neural networks [31]. Evaluation with DICOM data and learning with clinical data such as tumor markers can also improve diagnostic performance. Further versatility can be achieved using the images obtained with other MRI equipment.

## Conclusions

In conclusion, deep learning demonstrated high diagnostic performance in diagnosing the presence of endometrial cancer on MRI. In particular, a deep learning model using convolutional neural networks showed significantly better results with the single image set of axial apparent diffusion coefficient of water maps and axial contrast-enhanced T1-weighted images compared to expert radiologists. Moreover, although there were no significant differences, the addition of other types of images to the training data improved the diagnostic performance for some of the single image sets.

## Data Availability

The datasets generated and analysed during the current study are not publicly available due to the security of data but are available from the corresponding author on reasonable request.

## Abbreviations

CNNs: Deep learning models using convolutional neural networks
T2WI: T2-weighted image
ADC: Apparent diffusion coefficient of water
CE-T1WI: Contrast-enhanced fat-saturated T1-weighted image
DWI: Diffusion-weighted image
DICOM: Digital imaging and communications in medicine
JPEG: Joint photographic experts group
ROC: Receiver operating characteristic
CI: Confidence interval
AUC: Area under the receiver operating characteristic curve
